# 13th meeting of the European Society for Traumatic Stress Studies in 2013: selected papers

**DOI:** 10.3402/ejpt.v4i0.23582

**Published:** 2013-12-20

**Authors:** Dean Ajdukovic, Miranda Olff

**Affiliations:** 1 Department of Psychology, University of Zagreb, Croatia; 2 Department of Psychiatry, Academic Medical Centre, University of Amsterdam & Arq Psychotrauma Expert Group, Diemen, the Netherlands

International researchers and clinicians with an interest in psychotrauma joint at the 13th meeting of the European Society for Traumatic Stress Studies (ESTSS) in 2013, in Bologna, Italy. In this cluster, we present a selected set of papers based on (preconference) workshops or symposia held in addition to a symposium organised by the ISTSS with experts on disaster psychosocial care, in collaboration with ESTSS.

The issue was edited by Prof Dean Ajdukovic and presents an interesting mix of papers, some directed at clinical practice and others addressing more theoretical issues.

Simonelli (2013) describes classification and diagnostic issues of posttraumatic stress disorder in early childhood. Several adaptations to the classical adult criteria have been suggested and are discussed in this article.

Two traumatic stress interventions are presented. Gersons and Schnyder (2013) describe the Brief Eclectic Psychotherapy for PTSD (BEPP) which is an evidence-based treatment that combines elements from various psychotherapy schools. The authors show how it is different from other trauma-focussed treatments like CBT and EMDR and how patients may learn from their traumatic experience. Robertson, Blumberg, Gratton, Walsh and Kayal (2013) describe a group-based approach to stabilisation and symptom management in a phased treatment model for refugees and asylum seekers.

Craparo, Schimmenti and Caretti (2013) present a study on a sample of violent offenders from Italy and how traumatic experiences in childhood relate to psychopathy, while in Ardino, Milani and Di Blasio (2013) a study is presented on PTSD and re-offending risk and the mediating role of worry and negative perception of other people's support.

Finally, in the article based on the ISTSS symposium by Reifels et al. (2013), lessons learned are presented about psychosocial responses to disaster and mass trauma for a total of six disasters that occurred in five countries.

*Dean Ajdukovic* Department of Psychology University of Zagreb Croatia *Miranda Olff* Department of Psychiatry, Academic Medical Centre University of Amsterdam & Arq Psychotrauma Expert Group Diemen, the Netherlands
